# Asthma diagnosis and treatment – 1002. FEF25-75%: a more sensitive indicator in the early detection of asthma

**DOI:** 10.1186/1939-4551-6-S1-P2

**Published:** 2013-04-23

**Authors:** Marzieh Tavakol, Mohammad Gharagozlou, Mohsen Afaride, Masoud Movahedi, Zahra Tavakol

**Affiliations:** 1 Allergy and Clinical Immunology, Tehran University Of Medical Sciences, Children Medical Center, Tehran, Iran; 2 ISAA, Cipla, India; 3 Tehran University of Medical Sciences, Children Medical Center, Tehran, Iran

## Background

Spirometry is widely regarded as a clinically invaluable measurement method that is of genuine recommend for the diagnosis and management of asthmatic patients. FEV-1 and FEV1/FVC are vastly perceived as asthma severity and control assessment indices, according to the present clinical guidelines. Since FEV-1 index is chiefly within normal ranges even in the most severe cases, the certain criteria for asthma diagnosis is immensely base upon it's significant alteration after bronchodilator challenge test . FEF25-75% represents a well-established indicator of small airway disease now for decades, however, and It has been demonstrated that asthmatic patients do have remarkably lower FEF25-75%. Additionally FEF 25-75% meaningful response to the bronchodilator challenge test is seen in some asthmatics that are healthy in terms of other prognostic parameters***.***

## Objective

This study was designed to detect the most sensitive index for the diagnosis of asthma and to determine the correlation between FEV-1 and FEF25-75% indices and asthma control questionnaire (ACQ) scores.

## Methods

we recruited 107 patients with the diagnosis of asthma who were attending follow-up sessions at the Children's Medical Center Hospital (CMCH) between December 1, 2010 and May 31, 2012 to conduct a hospital- based study. A p value if <0.05 was considered to be clinically significant to our study.

## Results

FEF25-75% Response proved to be more sensitive in detection of asthma in comparison to FEV-1 Response as shown in our study ( p= 0.042 ). Nevertheless, Pre-bronchodilator FEF 25-75% does not follow this trend, compared to Pre-bronchodilator FEV-1 (p = 0.69). FEF 25-75% Response and ACQ score were significantly correlated (p= 0.01) while this is not the case between FEV-1 Response and ACQ score (p= 0.46). Moreover, Pre- bronchodilator FEF 25-75 had a meaningful relationship with ACQ score (p= 0.03), as opposed to the pre-bronchodilator FEV-1 in which no significant correlation was seemingly spotted (p= 0.17).

## Conclusions

FEF25-75 % provides a more sensitive way to assess the early detection, severity and progression of asthma, contrary to the conventional FEV-1 index that currently constitutes the only certain clinical criteria to fulfill this role.

